# Growth and inequality trade-offs to eradicate absolute poverty

**DOI:** 10.1016/j.heliyon.2023.e21441

**Published:** 2023-10-23

**Authors:** Jihoon Min, Narasimha D. Rao

**Affiliations:** a International Institute for Applied Systems Analysis (IIASA), Schlossplatz 1, A-2361, Laxenburg, Austria; b Yale School of the Environment, Yale University, New Haven, CT, 08511, USA

## Abstract

The relationship between growth, inequality and poverty remains elusive, despite considerable scholarship. To what extent can governments rely on growth to eradicate poverty without reducing inequality? We derive a closed-form relationship between a minimum income threshold, changes in the Gini index of income inequality and average national income required to meet this target, independent of the form of income distribution. We develop a generalized form of redistribution and validate it against historical changes in inequality. We use this formulation to illustrate feasible growth-redistribution strategies to raise entire populations above the International Poverty Line ($1.90/day) by 2030, the Sustainable Development Goal 1, in two selective countries: India and Rwanda. We show that meeting this target would require unprecedented rates of both growth and inequality reduction in Rwanda. India could not eradicate acute poverty by 2030 with growth alone, but it could with only a modest reduction in inequality.

## 1 Introduction

Poverty eradication has been understood as ensuring that everyone has access to a minimum level of resources. The Sustainable Development Goal 1 (SDG1) aims to eradicate extreme income poverty, which entails raising everyone's income to the World Bank's International Poverty Line (IPL) of $1.9 per day by 2030. The idea of a minimum income threshold has gained prominence even in wealthy countries, popularly known as a Universal Basic Income (UBI). Such a goal can ultimately be achieved through some combination of economic growth and income redistribution. However, its attainment is constrained by practical limits to accelerating growth and reducing inequality, particularly in resource-constrained least developed countries.

Governments have largely focused on growth as a vehicle for poverty eradication. The experience of China pulling hundreds of millions out of poverty is widely touted as a success of growth-oriented development. However, the replicability of the Chinese experience cannot be taken for granted, considering that even in China previous high growth rates seem to be no longer sustainable. In India, which just became the most populous country, growth has contributed to reducing extremely poverty from although acute poverty from almost half the population in 1990 to 10% in 2019 [1]. However, the share of people below a higher threshold of $5/day, which was still 84% in 2019 [2]. There are also millions who fall in and out of extreme poverty depending on economic conditions [3]. These factors point to the importance of reducing structural inequality to sustain reductions in poverty at higher levels of income.

Given the increasing attention to inequality and the disproportionate share of income growth accruing to the very wealthy [4,5], redirecting this excess through progressive taxes to the poorest would be appealing. While there are several examples of pro-poor social programs or other redistributive measures, the use of extreme wealth to finance them is not always apparent or politically acceptable. Governments often provide financial relief in times of crisis, such as during Covid, to all or some subset of citizens through flat payments, which are inherently progressive in their impact [6]. Thus, the relationship of poverty alleviation to inequality reduction is not easily discerned.

There has been substantial academic interest in the more general relation between growth, inequality and poverty [[7], [8], [9], [10]]. However, most studies focus on the interplay between two of the three variables, such as Kuznet's famous curve relating growth and inequality, or the influence of growth on poverty. Bourguignon's “Poverty-Growth-Inequality Triangle” is a rare exception that qualitatively lays out the importance of considering redistribution and growth together in examining poverty eradication. He notes from past experience that the influence of changes in inequality on poverty can offset that of growth [11]. A subsequent empirical work decomposes the influence of each pair of variables on the outcomes of the third, further corroborating this point [12]. Empirical works on this relationship treat poverty as an outcome variable, influenced by changes in growth and/or inequality conditions. Hence they focus on deriving or simulating growth elasticity of poverty and/or how it relates to inequality changes [[13], [14], [15], [16], [17], [18], [19]]. Lackner et al. for example, simulate the impact of various assumptions of Gini and growth rates on poverty headcounts based on a fixed poverty level (i.e. $1.9 per day) [17]. This is a natural approach given the reduction of poverty as a typical policy target of “growth-oriented” and “equity-oriented” development policies [20,21].

Most academic works relating the UBI and its implications to inequality either remain at the qualitative level [22,23] or examine particular UBI schemes designed for certain countries (mostly European cases) and how the schemes will change the base-year income distribution and Gini index [[24], [25], [26]].

Some studies investigate whether countries have enough capacity to eradicate their own poverty gaps by introducing marginal tax rates for non-poor population [27,28]. While they share similar viewpoints on the challenges of domestic redistribution in meeting the poverty target, they don't explore quantitatively the role of economic growth in tackling the same target.

We see that there is still a gap in the literature in understanding quantitatively and normatively what combinations of growth and redistribution can fully achieve a given target of poverty eradication. This inquiry is important to estimate how far different countries are situated from the SDG goal, and thus how much collective effort would be needed to eradicate global poverty.

We present a generic closed-form formulation, which relates any given minimum income threshold to changes in a national Gini index and average income (GDP) growth, independent of the form of income distribution. We define a non-exhaustive but expansive set of distributional transformations to simulate the provision of a minimum income under different combinations of growth and inequality changes. Our goal with this formulation is to assess the feasibility of eradicating poverty considering historical trends in income Ginis and growth in each country. This work and the suggested formulation are intended for poverty eradication in the absolute sense, which means reducing the headcount below a certain absolute income threshold. Throughout the work, we adopt the World Bank's global poverty lines as the basis of the analysis, but the formulation itself is agnostic about quantities or thresholds.

We apply our method to Rwanda and India, as illustrative examples of economies with high poverty headcounts, but with significant differences in capacity for redistribution, as indicated by the size of their economies, average wealth and extents of industrialization (Table 1). While both have quite high economic growth rates, India has a substantially higher average income and lower income inequality than Rwanda. Low income and higher Gini for Rwanda mean much higher poverty headcount ratios. According to the World Bank's country groupings, Rwanda belongs to “low-income” group ($1045 GNI per capita or less), while India to “lower-middle income” group ($1046 to $4095).

**Table 1:** Baseline information for India and Rwanda. Gini and Palma values are the latest available estimates for each country and based on consumption expenditure at the time of calculation. Values are for the year 2016 unless otherwise noted in parentheses. Actual income Ginis for India are above 50 [29]. Data source: World Bank, WIID, and [30].

2016 values	India	Rwanda
GDP/cap ($2017 PP P)	$5790	$1908
Growth rate (average 2006–16)	5.4 %	4.8 %
Gini	37.8 (2011)	43.7
Palma ratio	1.63 (2011)	2.25 (2017)
Poverty headcount ratio ($1.90/day)	18.1 %	52 %

We ask the following questions: can India and Rwanda grow their way out of poverty by 2030, consistent with the SDGs? How close to or far away from the required combinations of growth and inequality reduction required to eradicate poverty would the countries be by 2030 based on historical trends? Different aspects take priority in each case. Given Rwanda's low average income, faster growth would be essential, but what level of growth would it need to meet SDG1? Given India has relatively high growth but increasing inequality, to which extent can reducing inequality relieve the pressure to maintain high growth rates?

**Fig. 1:**
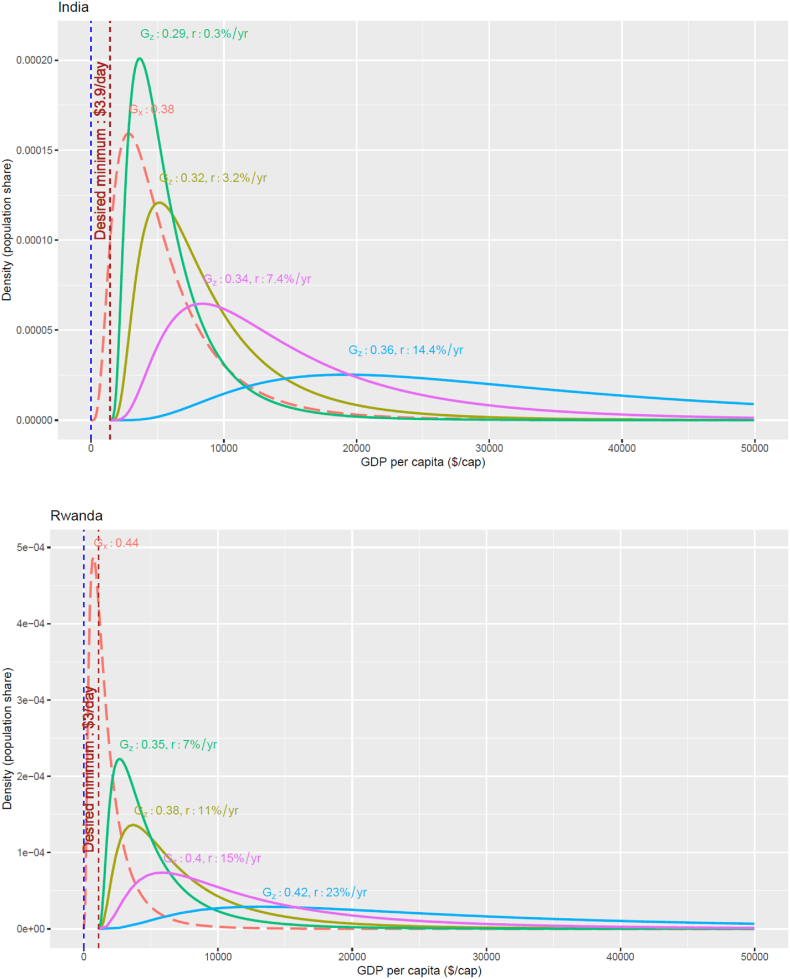
Illustrative target distributions for India and Rwanda in 2030 for combinations of Gini (G_z_) and average annual per-capita GDP growth (r) that achieve a minimum GDP/cap of $3.9/day (2017$ PPP) by 2030. These examples assume lognormal shaped distributions for the illustration purpose of the affine transformation. The orange dotted lines are for the base year 2016.

We examine an expansive set of Gini/growth combinations by representing income redistribution as an affine transformation (AT) of income—scaling and shifting the income distribution to achieve a minimum threshold. In practice, this is akin to providing a flat payment to all citizens, financed by a flat tax. This can be interpreted as a different parametrization of the Lorenz-convex transformation used by Ferreira and Leite [31]. This transformation can capture both growth- and equity-oriented distributional changes ranging from the extreme growth case, where growth is solely relied on to achieve the minimum (while keeping the constant Gini), as well as certain patterns of no-growth mean-preserving transfers. Fig. 1 below illustrates how these affine transformations achieve the minimum thresholds for different growth/redistribution combinations, using the lognormal function for illustrative purposes.

The main feature of the AT is that it is distribution-neutral (i.e. the relative distance is preserved) [31,32]. This entails that all the variations of redistribution that can be implemented to spread the burden of redistribution across the entire population. We justify the use of the AT as a generalized form of redistribution by examining historical income or consumption distribution data (by decile) of various countries. We estimate the best-fit affine transformations over time and show that this transformation can reasonably mimic distributional changes observed in each country for the last few decades (Section 3.1).

As mentioned earlier, there are other ways to selectively distribute the burden of redistribution across the income distribution. As a way to exemplify an extreme equity-oriented policy, we compare a separate ‘top-to-bottom’ redistribution scheme (TB) with no growth, where we transfer the income from the richest quantile to the poorest until everyone meets the minimum. However, in order to implement this scheme, we do have to assume a specific form of distribution, unlike in the general case of AT, because the AT formulation does not apply for the TB case. We pick the lognormal distribution due to its popularity and convenience, since the base year distribution can be estimated from just the observed mean and the standard deviation (which directly connects to observed Gini). Aside from this caveat, with these two redistribution schemes, AT and TB, we capture a wide range of combinations of GDP growth rates and Gini coefficients required to achieve SDG1.

We use two inequality metrics: Gini index and Palma ratio (the share of the richest 10 % divided by the share of the poorest 40 %) to capture distributional changes in inequality overall and at the extremes. Both are measures of relative inequality. The selection of these metrics is based on two considerations. First, the two metrics are very widely used in inequality analyses and therefore easily understood. Second, they nicely capture different aspects of inequality—Gini represents the overall dispersion of the distribution, while Palma focuses on the ratio of the tails of the distribution, thereby showing the contrast between the richest and the poor more effectively.

There are other perspectives on inequality (i.e. absolute, relative, or intermediate views), including how people perceive inequality differently [[33], [34], [35]]. While other approaches have merit, we adopt the relative measures partly because they are well established [36]. In any case, we note that the transformation (AT) we suggest here (Fig. 1) can represent both relative and absolute aspects of inequality.

## 2 Methods

### 2.1 The analytical framework

We re-parametrize the formulation by Ferreira and Leite [31] to directly represent the impact of growth and redistribution that would raise the minimum income of the population above a certain level (e.g., UBI). We start with an initial distribution of income X: $F_{X}$. A generic affine transformation of X gives Z=kX+d. The factor k represents a uniform scaling of the entire distribution, while d shifts all incomes by a fixed amount, which enables the provision of the minimum level. Comparing to Ferreira and Leite's ‘tax and benefit’ scheme, it means k=(1−α)(1+β) and $d=\alpha \left(1+\beta \right)\mu_{X}$, where β is the mean income growth rate, α is the hypothetical tax rate which then become a Gini improvement rate, and $\mu_{X}$ is the mean income per capita. As we focus on the achievement of zero poverty target and the corresponding poverty line, we isolate *d* in the formulation to indicate that everyone is above that minimum threshold *d* after the goal is achieved.

This reparameterization formulates the trade-off between growth and inequality more directly.

The linear transformation indicates that $\mu_{Z}$, the mean of Z, is

(1) $$\begin{array}{c} \mu_{Z}=k\mu_{X}+d=\left(1+\beta \right)\mu_{X}\text{.} \end{array}$$

With Equation [1], Equation [4] of Ferreira and Leite [31] becomes

$$G_{Z}=\left(1-\alpha \right)G_{X}$$

(2) $$\begin{array}{c} =\frac{k}{\left(1+\beta \right)}G_{X} \end{array}$$

$$=\frac{k\mu_{X}}{k\mu_{X}+d}G_{X}\text{.}$$

To raise the entire population above a minimum income level D with this affine transformation, we can define the relationship between *k, d,* and *D* as:

(3) $$\begin{array}{c} d=D-k\cdot \min\left(X\right)\text{.} \end{array}$$

We can then replace *d* in Equation [2] with [3]:

$$\begin{array}{c} G_{Z}=\frac{k\mu_{X}}{k\left(\mu_{X}-\min\left(X\right)\right)+D}G_{X}\text{.} \end{array}$$

We see when we can assume min(X)=0, simply d=D.

Depending on the relative sizes of parameters k and d and the sign of d, it can represent a wide range of realistic changes in distributional patterns. It can capture a change involving an income growth at some quantiles with a decrease in others such as income polarization with a shrinking middle class. Mean-preserving progressive or regressive transfers can also be captured when $\mu_{Z}=\mu_{X}$. When d is strictly positive, poverty reduction is accompanied by improving inequality (i.e. decreasing Gini), which also has empirical support [18,37].

Also note that the relative sizes of $\mu_{X}$ and $\mu_{Z}$ depends on k and d, so this transformation can represent either positive or negative growth of an economy. When we assume this transformation takes n years, the annual per-capita income growth rate $r_{inc}$ enabling this transformation can be derived in a straightforward manner. And this directly relates to the parameter β in Ferreira and Leite's formulation [31].

$$\begin{array}{c} r_{inc}=\left(\sqrt[{\frac{1}{n}}]{\frac{\mu_{Z}}{\mu_{X}}}-1\right)={\left(1+\beta \right)}^{\frac{1}{n}}-1 \end{array}$$

One could additionally account for average annual population growth rate $r_{pop}$ in this period to get necessary annual national income growth rate.

$$\begin{array}{c} r_{nat}≅r_{pop}+r_{inc}\text{.} \end{array}$$

In sum, from this formulation, with a given 3-tuple of $(G_{X}$, min(X), $\mu_{X})$ at the base year and a desired minimum resource threshold D to be achieved for the whole population in n years, we can define a distribution-neutral solution space for ($G_{Z},r_{nat})$ or ($G_{Z},r_{inc})$. Note that although the transformation is distribution-neutral, the mathematical formulation of the distribution before and after the transformation may change. For instance, when d is a non-zero value, a usual two-parameter lognormal (which assumes the minimum value of zero) becomes a three-parameter lognormal often used in hydrology [38]) after the transformation, but a Gaussian would still remain Gaussian.

To implement the top-to-bottom (TB) case, we first look for a level in the distribution, the sum of the income above which will match the poverty gap for all people under the minimum threshold. And the entire amount above this level is transferred to the poor (i.e., 100 % tax rate applied to the wealth above the line). Given that we do not have enough information about the income distribution at the top, this TB calculation cannot be done with existing data. Here we estimate lognormal distributions from the historical parameters, which is commonly adopted to model income distribution and is empirically found to represent most of low-mid income population [39]. Our TB scheme is a bit different from Bolch et al.‘s tax scenario 2 [28], which applies a fixed tax rate for population above a given affluence line ($13/day (2005 PPP)) to fill the poverty gap based on a different poverty line ($2/day (2005 PPP)). So, their results are not directly comparable with our findings.

### 2.2 Data

To illustrate this framework, we collected information from multiple data sources. The historical inequality data (historical Gini and Palma ratio) are mainly taken from the World Income Inequality Database (WIID) and cleaned to make them internally consistent [30,40]. And some missing observations not found in the WIID are looked up from the World Bank's PovcalNet [41]. We rely on the WIID also for the historical decile shares of household income or consumption by country for the validation of our assumptions. The World Bank's WDI (World Development Indicators) database was referred to for various country-specific economic indicators, such as historical GDP per capita, economic growth rates, poverty headcounts. These data sources are all publicly available, but we can also share the result calculations upon request.

## 3 Results and discussion

### 3.1 Empirical validity of the affine transformation

We examine how well the affine transformation captures historical distributional changes, using the timeseries of decile shares of household income or consumption by country from the WIID. We construct Lorenz curves for several sample countries (Fig. S1), including India and Rwanda, having relatively larger number of observed years from consistent data sources. For each country and each year, we find the best-fit Lorenz curve $L_{X,y,AT}$, affine-transformed from the base-year Lorenz curve $L_{X,y_{0}}$, which is the closest (based on the least-square method) to the observed Lorenz curve $L_{X,y}$. Since a Lorenz curve $L_{X}$, for any given year, is an integrated form of a quantile function $F_{X}^{-1}$ as established in Ref. [42],

$$L_{X}\left(q\right)=\frac{\int_{0}^{q}F_{X}^{-1}\left(p\right)dp}{\mu_{X}}$$

The affine transformation on X means the same transformation applied vertically to the given Lorenz curve of X. So that means, for each year y, we find an affine transformation $Z=k_{y}X+d_{y}$ which will minimize the sum of squares of the distances between $\left\{l_{i,y}\right\}$ and ${\{l}_{i,y,AT}\}$, the decile points of $L_{X,y}$ and $L_{X,y,AT}$ curves (i: decile index), i.e.

$$\min\sum_{i=1}^{10}{\left(l_{i,y}-l_{i,y,AT}\right)}^{2},s.t.\;l_{i}=l_{i,AT}=1$$

**Fig. 2:**
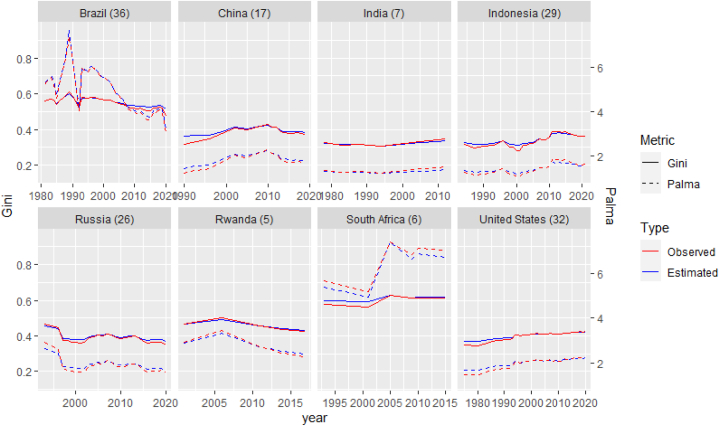
Comparing the observed and the estimated inequality metrics: Gini and Palma ratio. The numbers in parentheses mean the number of observation years for each country. The two timeseries remain close to each other for the decades they were observed. (Own calculation).

The base year $y_{0}$ to apply the affine transformation to is chosen around the midpoint of the observed range of years, to more evenly distribute/visualize the errors on both sides of the base year. We compare two inequality metrics—Gini and Palma ratio—for the observed and the estimated (i.e., affine-transformed) curves (see Fig. 2). We find, for many decades of observations, the affine transformation tracks reality for both inequality metrics. Overall, the deviations seems to be within a reasonable range. About 95 % of Gini estimates deviate within 2.2% points from the observed values. The largest Gini difference is around 5% points for China in 1990. These findings suggest that we can expect the AT to be useful in projecting the future development of inequality for most economies.

Despite this agreement with reality, this transformation cannot represent all, particularly short-lived, structural changes in the distributions. Moreover, even though the aggregate distributional change, as reflected in the inequality metrics, can be approximated by the AT formulation, it does not necessarily mean that the real-life redistribution always follows the AT.

### 3.2 Growth/inequality combinations to achieve SDG1

We illustrate the value of our framework using India and Rwanda as examples, because they represent very distinct starting points (see Table 1). We choose the World Banks acute poverty threshold ($1.90/day) target in SDG1 for quantifying absolute poverty in the two countries. As this threshold is defined in household income or expenditure terms, we convert it to 2017$PPP GDP per capita using household income shares out of GDP for the two countries (60% for India and 70% in Rwanda) [43] PPP, which works out to $3.9/day (India) and $3.0/day (Rwanda) in GDP terms.

**Fig. 3:**
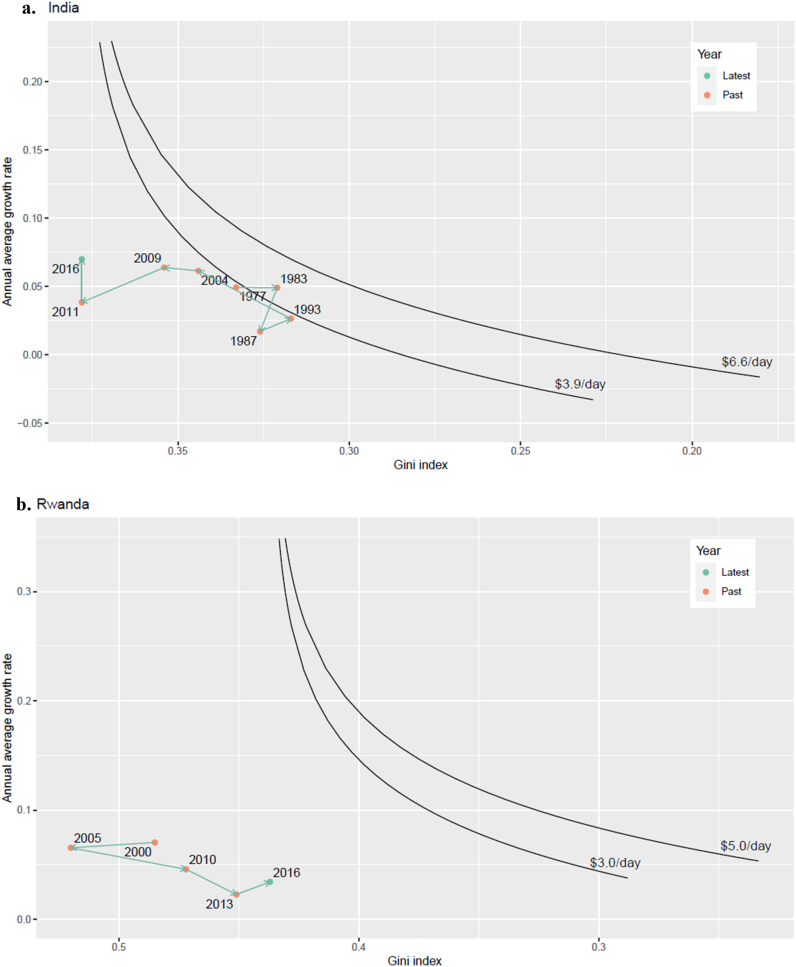
Combinations of Gini index (x-axis) and per-capita GDP annual growth rates to achieve different minimum income thresholds by 2030, shown in terms of GDP/capita, for a) India and b) Rwanda. Colored dots show historical combinations of Gini/growth rates. The household income shares among GDP is assumed 60% for India and 70% for Rwanda. At the time of this calculation, Gini for India is not reported for 2016 by WIID, so we assume it is unchanged from 2011.

We visualize, in Fig. 3, solutions for ($G_{Z},r_{inc})$ (the black curves)—trajectories of required annual per-capita income growth rates ($r_{inc}$) and Gini index ($G_{Z}$—for the countries to achieve these target minimum income thresholds by 2030 (SDG1). This figure has a similar motivation to the isopoverty curve of Ferreira and Leite [31], but we plot annual growth and Gini to make the interpretation easier. The connected dots in the figure represent historical observations of the GDP growth rates and the Gini values in each country. A country needs to ‘reach’ any point on the black line from the latest observation point to secure the corresponding minimum for everyone. Reaching the curve through a vertical shift means growth-driven poverty eradication, while a horizontal movement is redistribution-driven, with various diagonal shifts representing different combinations of growth and redistribution. Note that these curves do not assume any forms of income distribution. One point asking for a cautious interpretation is that the growth rates on the curves are the average growth rates that need to be sustained until the target year, while the Gini values are the final values that can be reached by the target year. Hence, while a country can gradually move towards the curve horizontally, it in a sense needs to leap vertically to get to a desired average growth rate, if the current growth rate is significantly lower than any desired points on the curve.

In order to estimate Palma ratio for the affine transformation (AT) case, we do need to assume a specific distributional form to calculate the impact of the redistribution on deciles. Thus, we adopt lognormal distributions again, as we've done for the TB calculation. We compare Gini values and Palma ratios before and after the AT redistribution is applied to the assumed lognormal distribution.

In general, the curve shapes are intuitively downward sloping, implying that with higher growth rates, less inequality reduction is required to achieve a given minimum, and vice versa. Table 2 compares the base-year growth and inequality statistics with the desired outcomes to meet the SDG1 goal by 2030.

**Table 2:** Summary of the results for the two countries to achieve $1.9/day target by 2030. Grey-shaded cells for Rwanda indicates the transitions that will require Gini improvement rates almost unprecedented in history (see Table 3). (Own calculation).

India		Top-to-bottom (TB) (lognormal)			Affine Transformation (AT)		
		Growth rate p.a.	Gini	Palma ratio (lognormal)	Growth rate p.a.	Gini	Palma ratio (lognormal)
Base year stats 2016–2017		5.4 %	0.38	1.63	Same as left		
SDG1 achievement by 2030	no growth	0.0 %	0.37	1.59	0.0 %	0.29	1.04
	current growth				5.4 %	0.33	1.31
	10 %/yr growth				10 %	0.35	1.44
	current Gini				>60 %	0.38	1.60
**Rwanda**		**Top-to-bottom (TB) (lognormal)**			**Affine Transformation (AT)**		
		**Growth rate p.a.**	**Gini**	**Palma ratio (lognormal)**	**Growth rate p.a.**	**Gini**	**Palma ratio (lognormal)**
Base year stats 2016–2017		4.8 %	0.44	2.25	Same as left		
SDG1 achievement by 2030	no growth	0.0 %	0.30	1.07	0.0 %	0.19	0.69
	current growth				4.8 %	0.31	1.19
	10 %/yr growth				10 %	0.37	1.44
	current Gini				>70 %	0.44	2.25

#### 3.2.1 Putting the two example countries in context

The results for India in Table 2 show that eradicating acute poverty ($3.9/day GDP p. c.) by 2030 would be virtually impossible without reducing inequality, requiring unprecedented annual growth rates over 10 years. Note that the growth with constant Gini implies that the income of all members increases proportionately. Considering that inequality in India has steadily risen and remained well above 0.35 for almost two decades suggests that a significant departure from past policies is likely required to eradicate poverty.

In Rwanda, the minimum income target ($3.3/day GDP p. c.) is even further out of reach. Unprecedented levels of growth (>10% p. a.) and inequality reduction (to less than 0.4) would be required by 2030 to eradicate poverty. In the last two decades, although inequality has been reduced, the Gini index has remained well above 0.4, and GDP growth rates have been below 8% p. a. We do a sensitivity test for 2050 (See SI), which shows that eradicating acute poverty is still a challenge in Rwanda, but not impossible—if it can maintain average annual growth rates of ∼8% and a reduction in the Gini to 0.42 by then.

#### 3.2.2 Comparing two redistribution schemes

The change in inequality required in the TB scheme tells a different story. With this scheme involving only redistribution without growth, India can raise the minimum income to $1.9/day by reducing the Gini from 0.378 to just 0.373. The Palma ratio, which is the top decile income as a share of the bottom 40 percentile, is 1.63 in the baseline in 2016, assuming a lognormal distribution. The $1.9/day goal can be met by reducing the ratio only to 1.59. The *smaller* necessary changes for the inequality metrics for the TB scheme than those for the AT with no growth reflect the relatively obvious fact that there is enough wealth among even a handful of individuals to bring everyone out of poverty. Consider that, according to the World Bank, the poverty gap (defined as “the mean shortfall in income or consumption from the poverty line”) in 2016 was 3.6 % (of $1.9/day) in India [44]. For ∼250 million people (poverty headcount of 18.7% in 2016), in GDP terms this is $10 billion ($2017PPP), which is less than the net worth of each of the top twenty billionaires in the world [4,45].^1^

Contrary to the smaller drops in inequality metrics for the TB case for India, Rwanda would still need to radically improve the inequality metrics to achieve the SDG goal (Gini 0.44 to 0.30 and Palma ratio from 2.25 to 1.07, Table 2). This is mainly because the majority of the population is in acute poverty in 2016. This rate of Gini improvement is almost absent in existing data observations (Table 3), which means in Rwanda's case combining unprecedented rates of growth and extreme redistribution would be the only options.

A more realistic scenario considers the necessary growth/redistribution combination from present levels of growth. In India, with 5.4% p. a. growth (2006–2016 average as shown in Table 1), the Gini would have to be reduced to 0.33 to eradicate the acute poverty ($3.9/day GDP p. c.). With a 10% average growth rate, the Gini would have to reduce to 0.35. This corresponds to the decrease of Palma ratio from 1.63 in 2016 to 1.31 (with 5.4 % growth) or 1.44 (with 10 % growth). The lesson here is that pursuing even modest efforts towards equitable growth reduces the burden on growth disproportionately. This is consistent with the general trend observed by Bourguignon and Khan [11,12].

The small inequality improvement for India for achieving the poverty target makes the TB case a desirable policy choice, but, in reality, hard to implement. The developing world's income in general has been increasing along with increasing within-country inequality [46], suggesting higher concentration of income at the top of the distribution. This makes the targeted redistribution attractive. Further, ideas of *universalism* and *targeting* in poverty eradication have been long-discussed topic [47]. However, such targeted redistribution to the poor at the bottom of the national distribution has not been successful in practice due to various political and administrative reasons [48,49]. Nevertheless, the TB case serves as an aspiration counterpoint to the AT, showing that shifts towards increasing progressivity through targeted redistribution can substantially reduce the burden of poverty eradication.

### 3.3 Historical pace of inequality reduction

Above we calculate necessary reductions in the Gini by the target year 2030. In Rwanda, due to lower average incomes, we see that present growth rates have to be significantly increased alongside aggressive redistributive policies to eradicate poverty. How realistic are these reductions in income inequality? We examined historical trends in the Gini data collected above, filtering out potential measurement errors by looking only at the 10-year spells containing at least four Gini observations for each country. We fit a least-square trend line to derive the Gini change rates to the filtered data. This leaves 65 spell observations over the overall period between 1954 and 2015, among which 60 observations have decadal average Gini changes within the range of ±10% point (p.p.)/decade (Table 3). The exceptionally fast Gini changes (10 p. p./decade or higher) might well be related to some politically or economically turbulent periods in corresponding countries, which we treat as exceptions to the norm. Lakner et al. [17] did a similar exercise, where they summarized the relative changes of Gini based on PovcalNet data and showed most of observations retain about ±2 % change per year. This translates to similar change rates to ours in p. p. for common Gini value ranges.

**Table 3:** The top 15 countries showing the fastest pace of Gini improvement. We select only those countries with at least four observations in a 10-year period to avoid potential measurement error. (Source: WIID and PovcalNet) The column “Gini@year0” means the Gini value of the country at the beginning of the 10-yr periods, and “# of obs” is the number of Gini data points during the 10-yr periods.

Country	Gini@year0	Period	Avg ΔGini/decade (p.p.)	# of obs
Serbia	0.393	2004–2013	−12.4	5
Venezuela	0.500	2002–2011	−11.6	10
Niger	0.444	2005–2014	−11.5	4
Bolivia	0.619	2000–2009	−11.5	8
Zambia	0.512	1993–2002	−9.8	4
Ghana	0.360	1988–1997	−9.7	6
El Salvador	0.518	1999–2008	−9.0	9
Slovakia	0.267	2002–2011	−8.8	7
Kyrgyzstan	0.489	1996–2005	−8.2	10
Cote d'Ivoire	0.452	1985–1994	−8.2	5
Kazakhstan	0.354	2001–2010	−7.6	10
Ukraine	0.352	1996–2005	−7.5	5
Iceland	0.286	2006–2015	−7.2	9
Chile	0.573	1999–2008	−7.1	4
Ecuador	0.559	2000–2009	−6.9	8

When we apply this to our formulation, we can derive empirical boundary conditions within the solution space shown in Fig. 3. With these inequality reduction restrictions, Rwanda will need to keep annual economic growth of 7.5% or higher until 2030 to achieve the poverty goal. If India were able to reverse past Gini trends and reduce inequality to the maximum extent of 10 p. p. by 2030 (from 0.38 to 0.28), India could eradicate the acute poverty ($3.9/day GDP p. c.) by 2030 even without growth.

In summary, this analysis confirms that for poverty eradication growth may be necessary in very poor countries, but it is an inefficient approach on its own in middle income countries like India without concomitant reductions in inequality. This has been pointed out in previous work, albeit only qualitatively. This study further provides a framework to estimate the reasonableness of SDG poverty eradication targets and realistic requirements for growth and redistribution. In Rwanda, given that unprecedented rates of growth and redistribution would be needed in the next decade, the SDG targets are not likely to be reached. Policymakers could project realistic poverty eradication targets using the results of this research combined with their own expectations of growth or inequality reduction. In India, we show inequality reduction is essential for poverty eradication. The AT and TB cases help define rough bounds on the effort required to eradicate poverty under different extents of progressivity in social policy.

Recent historical trends and their analysis by Piketty and others make clear that inequality persists due to structural characteristics of modern economies that favor wealth accumulation. Without fundamental changes in these structures, one has to face the sobering prospect that simple redistributive schemes like our TB case may be as unrealistic as they may be desirable. In that scenario, broad-based, structural redistributive mechanisms may be the only way to less the burden of growth. Our AT case is an attempt in this direction. Further research is needed to better relate actual redistributive policies to these mathematical representations. It remains a challenge to develop mathematical representations for inequality reduction that define the full possibility space, let alone those that resemble the real world.

## 4 Conclusion

We have derived a quantitative relationship between GDP growth rates and income inequality changes, as reflected in national Gini coefficients, to eradicate absolute poverty. The derivation was based on an observation that historical distributions of income or consumption can be closely mimicked by affine transformations, i.e., scale and shift the income distribution. The results emphasize the importance of considering both growth- and redistribution-oriented policies to eradicate poverty. We have looked at India and Rwanda, countries with very different levels of income and poverty headcounts, but similar levels of inequality. Both countries are unlikely to be able to grow themselves out of even acute poverty (World Bank's $1.90/day) by 2030. For India, modest changes in inequality reduce the required growth rates disproportionately. Least developed countries like Rwanda require both growth and redistribution at unprecedented paces—neither on its own can lead to poverty eradication even by 2050.

In this study, we have not considered the major dip in the world's economic growth in 2020 due to COVID19. In addition, the world is now going through an energy crisis caused by the Russian invasion of Ukraine. Both crises are ongoing and will likely exacerbate poverty and inequality around the world [50,51]. When these are considered, the annual growth and inequality improvement rates we would need to achieve SDG1 will likely have to be even higher than our estimates.

The strength of this formulation is that it does not depend on specific distributional assumptions and can be applied to any quantities other than income or consumption, and to any country or regions as long as the data allows. Policy makers may use this formulation to examine their poverty reduction targets. Further research could, for example, apply this formulation to assess the feasibility of other SDGs such as energy, water, etc., if their achievement can be linked to income changes. Studies of distributional impact of policies, such as in integrated assessment of climate change, may adopt this formulation to limit scenarios of growth and inequality to feasible combinations or to those that do not push people below a minimum threshold of poverty.

## Data availability statement

This research relies on publicly available data from the World Income Inequality Database (WIID) or the World Bank's PovcalNet. Hence there is no additional dataset deposited on a public repository by the authors. But we can provide any intermediate data or source codes for the calculations in the manuscript on request.

## CRediT authorship contribution statement

**Jihoon Min:** Conceptualization, Formal analysis, Investigation, Methodology, Software, Validation, Visualization, Writing – original draft, Writing – review & editing. **Narasimha D. Rao:** Investigation, Writing – original draft, Writing – review & editing, Data curation.

## Declaration of competing interest

The authors declare that they have no known competing financial interests or personal relationships that could have appeared to influence the work reported in this paper.

## Footnotes

1 As a comparison, for the ‘no growth’ case for the Indian AT case, we find the necessary Gini change is a 24 % decrease from 0.38 to 0.29 from Table 2 (α=0.24 based on Equation (2)). This indicates about $1.9 trillion ($2017PPP) needs to be moved around within the entire population to achieve the poverty goal through the AT redistribution. But at 10 % growth rate assumed, the amount comes down to $0.6 trillion.
