# Supplementary material for: Growth and inequality trade-offs to eradicate absolute poverty
Source: Heliyon. 2023 Oct 23;9(11):e21441. doi: 10.1016/j.heliyon.2023.e21441 (PMC10641217; doi:10.1016/j.heliyon.2023.e21441)
Supplement: Multimedia component 1 [file mmc1.docx]

## Supplementary Information
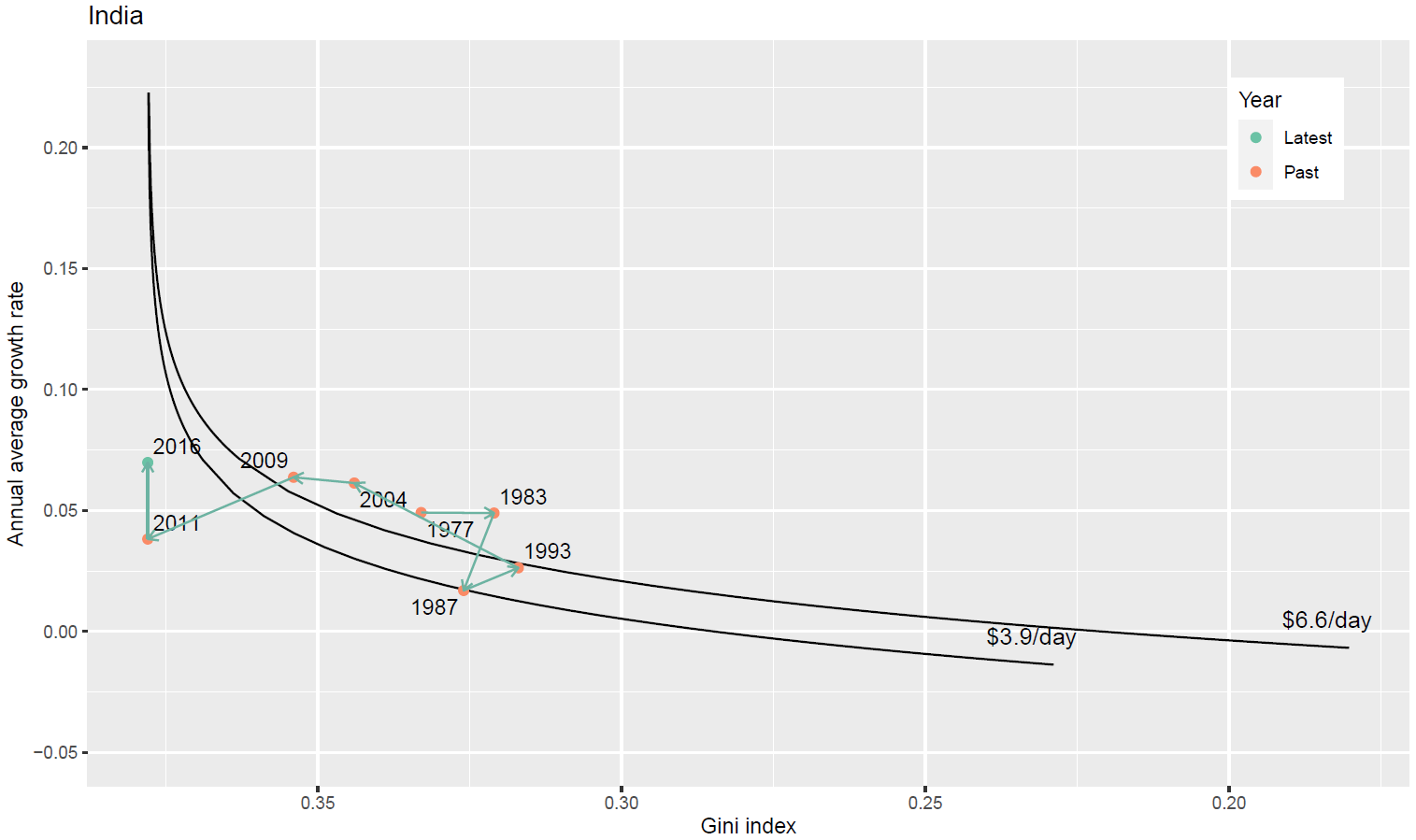

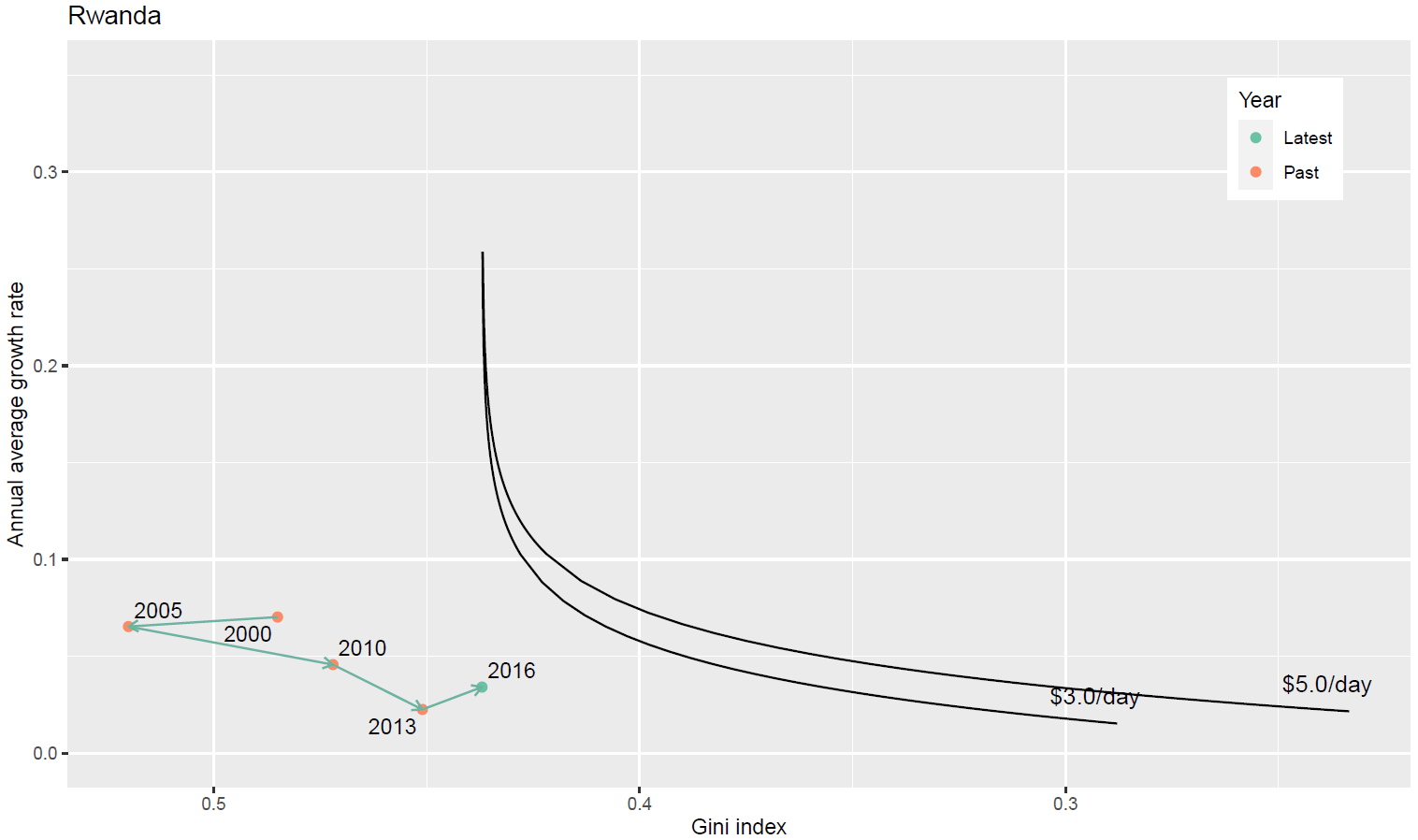


Figure S1. Combinations of Gini index (x-axis) and per-capita GDP annual growth rates to achieve different minimum income thresholds by 2050. Annotations are identical to Figure 1. Curves are lowered closer to the historic observations, which means that to achieve the poverty goal becomes less burdensome for those countries with the extended time window.


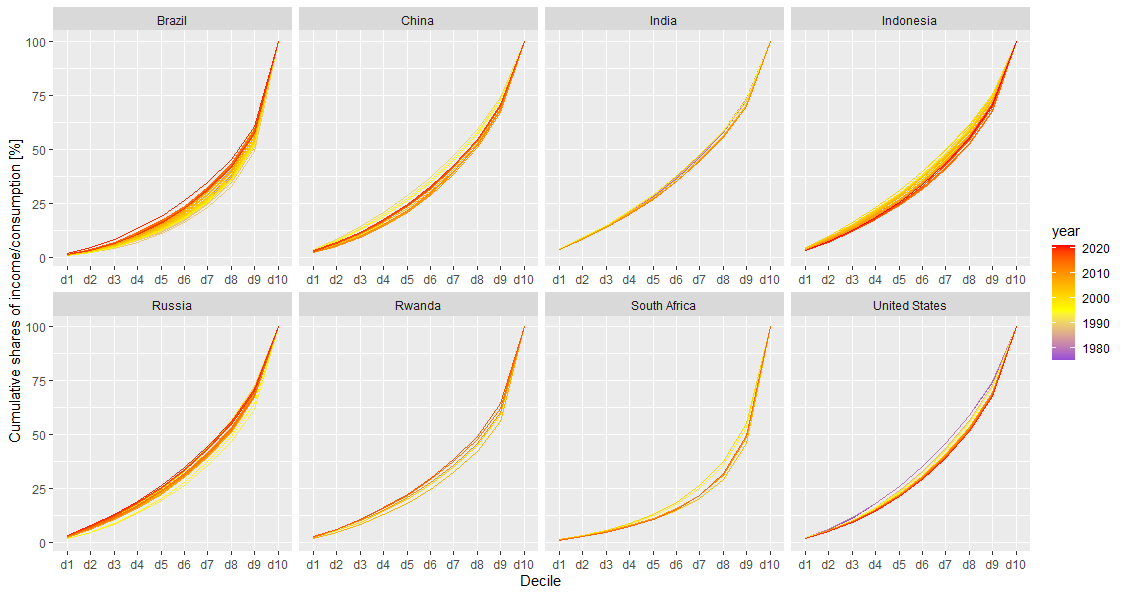


Figure S2. Lorenz curves of the selected countries over time.
